# S220. BLONANSERIN AUGMENTATION IN PATIENTS WITH SCHIZOPHRENIA – WHO IS BENEFITED FROM BLONANSERIN AUGMENTATION? AN OPEN-LABEL, PROSPECTIVE, MULTI-CENTER STUDY

**DOI:** 10.1093/schbul/sby018.1007

**Published:** 2018-04-01

**Authors:** Won-Myong Bahk, Young Joon Kwon, Bo-Hyun Yoon, Sang-Yeol Lee, Kwanghun Lee, Duk-In Jon, Moon Doo Kim, Eunsung Lim

**Affiliations:** 1 Catholic University of Korea; 2 College of Medicine, Soonchunhyang University; 3 Naju National Hospital; 4 Wonkwang University, School of Medicine; 5 College of Medicine, Dongguk University; 6 College of Medicine, Hallym University; 7 School of Medicine, Jeju National University; 8 Shinsegae Hospital

## Abstract

**Background:**

Evidences for antipsychotic augmentation for schizophrenic patients with sub-optimal efficacy have been lacking although it has been widespread therapeutic strategy in clinical practice. The purpose of this study was to investigate the efficacy and tolerability of blonanserin augmentation with an atypical antipsychotics (AAPs) in schizophrenic patients.

**Methods:**

A total of 100 patients with schizophrenia partially or completely unresponsive to treatment with an AAP recruited in this 12-week, open-label, non-comparative, multicenter study. Blonanserin was added to existing AAPs which were maintained during the study period. Efficacy was primarily evaluated using Positive and Negative Syndrome Scale (PANSS) at baseline, week 2, 4, 8, and 12. Predictors for PANSS response (≥20% reduction) was investigated.

**Results:**

The PANSS total score was significantly decreased at 12 weeks after blonanserin augmentation (-21.0 ± 18.1, F=105.849, p<0.001). Response rate on PANSS at week 12 was 51.0%. Premature discontinuation was occurred in 17 patients (17.0%) and 4 patients among them discontinued the study due to adverse events. Nine patients experienced significant weight gain during the study. Response to blonanserin augmentation was associated with severe (PANSS>85) baseline symptom (OR=10.298, p=0.007) and higher dose (>600mg/day of chlorpromazine equivalent dose) of existing AAPs (OR=4.594, p=0.014).

**Discussion:**

Blonanserin augmentation improved psychiatric symptoms of schizophrenic patients in cases of partial or non-responsive to an AAP treatment with favorable tolerability. Patients with severe symptom despite treatment with higher dose of AAP were benefited from this augmentation. These results suggested that blonanserin augmentation could be an effective strategy for specific patients with schizophrenia.

